# Hair cortisol concentrations in pregnant women with bipolar, depressive, or schizophrenic spectrum disorders

**DOI:** 10.1007/s00737-024-01434-4

**Published:** 2024-02-03

**Authors:** Maja Nyström-Hansen, Marianne Skovsager Andersen, Kirstine Agnete Davidsen, Katrine Roehder, Christopher Trier, Emilie Nayberg, Karlen Lyons-Ruth, Susanne Harder

**Affiliations:** 1 Department of Psychology, University of Copenhagen, Oester Farimagsgade 2A, 1353 Copenhagen, Denmark; 2 Department of Endocrinology and Metabolism, Odense University Hospital, Odense, Denmark; 3 Department of Psychology, University of Southern Denmark, Odense, Denmark; 4 Research Unit, Department of Child and Adolescent Mental Health Odense, Mental Health Services in the Region of Southern Denmark, Odense, Denmark; 5 Department of Psychiatry, Harvard Medical School, Cambridge Hospital, Cambridge, USA

**Keywords:** Hair cortisol, Pregnancy, Bipolar disorder, Depressive disorder, Schizophrenia, Symptomatic functioning

## Abstract

**Purpose:**

Maternal cortisol levels in pregnancy may support the growth of or adversely affect fetal organs, including the brain. While moderate cortisol levels are essential for fetal development, excessive or prolonged elevations may have negative health consequences for both the mother and the offspring. Little is known about predictors of altered hypothalamic–pituitary–adrenal (HPA) axis activity during pregnancy. This study examined maternal hair cortisol concentration (HCC) in the 3rd trimester of pregnancy in relation to severe psychopathology.

**Methods:**

Hair samples were collected from 69 women, 32 with a lifetime diagnosis of severe mental disorders (bipolar I or II disorder, moderate or severe depressive disorder, schizophrenic spectrum disorder), and 37 non-clinical controls. Hair samples were collected during the 3rd trimester, and liquid chromatography tandem mass spectrometry was used for cortisol assessment. Psychiatric diagnosis and current level of symptomatic functioning were assessed using the structured clinical interview from the DSM-5 and the global assessment of functioning scale.

**Results:**

Women with a lifetime diagnosis of severe mental illness had significantly elevated HCC compared to controls. Poorer current symptomatic functioning was also significantly associated with elevated HCC in pregnancy.

**Conclusions:**

The implications of alterations in HCC on both maternal and infant health need further study.

## Introduction

A large body of research has established an intergenerational transmission of risk of psychopathology in offspring of parents with severe mental illness (SMI), such as bipolar disorder, psychosis, and depression (Bifulco et al. 2002; Willcutt and McQueen 2010; Uher et al. 2014; Liu et al. 2015). Meta-analytic findings indicate that the heritable risk of developing SMI is best perceived as a general increased risk for any mental disorder more than a diagnosis-specific risk (Rasic et al. 2013). Recently, dysregulation of the human stress regulatory system has been suggested as a potential common factor across various SMIs that may be involved in the intergenerational transmission of psychopathology risk from mother to offspring.

The prenatal period is a time of elevated vulnerability to stress for the mother-to-be (reviewed in Khoury et al. 2023). Furthermore, growing evidence suggests that maternal stress during pregnancy can have long-term effects on fetal development (Zijlmans et al. 2015). Prenatal stress may lead to long-term changes in offspring regulation of the hypothalamic–pituitary–adrenal axis (HPA) as well as other negative developmental and health outcomes of the offspring (De Weerth and Buitelaar 2005; Van den Bergh et al. 2020; Andreasen et al. 2023). As such, the effect of prenatal stress could constitute an important risk factor for developing psychopathology later in life.

Several studies have assessed the change in hair cortisol concentration (HCC) levels during pregnancy and found a consistent pattern of circulating cortisol increasing through pregnancy (reviewed in Mustonen et al. (2018)). Maternal cortisol levels triple in the 3rd trimester (Jung et al. 2011), and the fetus is more exposed to the cortisol as the enzyme activity of 11beta HSD 2 is reduced in the 3rd trimester (Seckl and Holmes 2007). This makes pregnancy an important time for exploring the status of the HPA axis in women with psychopathology (Wikenius et al. 2016; Broeks et al. 2021).

HPA axis activity may be assessed by serum, saliva, or urinary cortisol, and recently, hair cortisol has emerged as a novel approach for measuring long-term cortisol exposure. A single hair sample provides an integrated measure of chronic cortisol production, where 1 cm of hair provides a measure of one month’s cortisol production (Kirschbaum et al. 2009). Hair cortisol levels in pregnancy can be a useful marker of overall maternal HPA activity during each trimester (Anna-Hernandez et al. 2011). In previous studies, primiparity was associated with higher cortisol levels compared to multiparous women (Andersen et al. 2019).

Psychopathology has been variously associated with both HPA axis hypo- and hyperactivity from samples outside pregnancy (Staufenbiel et al. 2013). Anxiety and PTSD are associated with decreased cortisol levels, major depressive disorder, bipolar disorder, psychosis, and schizophrenia have been linked with increased HCCs (Staufenbiel et al. 2013; Borges et al. 2013). However, we still need more knowledge of HPA axis functioning in people with severe psychopathology especially during the sensitive time of pregnancy. The existing research yields inconsistent information on the associations between self-reported symptoms of psychological distress, such as depressive symptoms, during pregnancy, and HCC axis functioning. In a systematic review, Mustonen et al. (2018) reviewed studies assessing the link between maternal psychological distress and HCC; two of five studies reported a positive association between maternal prenatal psychological distress and HCC, and three reported no association. Mustonen et al. (2018) concluded that HCC appeared to be inconsistently associated with self-reported symptoms of prenatal psychological distress, especially in the range of mild-to-moderate symptom levels, and that characteristics of the study population and study design might partly explain whether associations between distress and HCC were observed. Self-reports of psychological distress usually cover short time periods (e.g., the last 1–2 weeks) that may not be closely related to HCC. Furthermore, all of the studies in the systematic review (Mustonen et al. 2018) included subjects from the general population with overall low levels of psychological distress and no data on psychiatric diagnoses.

A recent meta-analysis by Khoury et al. (2023) supplement these findings. Though the overall meta-analysis showed a non-significant effect between psychological distress and HCC, Khoury and colleagues (2023) report that studies using self-reported stress and depression showed significant associations between psychological distress and HPA activity. However, when stress was further subcategorized, only studies that assessed more chronic life stressors and not those that measured perceived stress or pregnancy stress were significantly associated with HPA axis functioning. In addition, moderator analyses indicated that the strength of the association between psychological distress and HCC was moderated by timing of HCC and distress measurement, so that effects were larger when distress was measured before HCC.

It seems that duration of stress more than certain types of psychological distress, related to mood, may be more strongly linked to HPA activity compared to more transient stressors. A Norwegian study assessing depressive symptoms during pregnancy did not find current symptoms to be associated with HCC in a community sample (Wikenius et al. 2016). They suggested that the construct of lifetime diagnosis might be more informative when evaluating the associations between HCC and psychopathology. Thus, including measures reflecting both long-term symptomatology as well as short-term psychological distress may be especially valuable in studies evaluating the association between maternal psychopathology and prenatal maternal HPA axis functioning.

The aim of this study is to assess psychopathology as a predictor of HCC in the 3rd trimester in pregnant women with severe mental disorders and non-clinical controls. We hypothesize that HCC is higher in individuals with a lifetime diagnosis of severe mental disorder compared to controls and that higher HCC is associated with ratings of poorer current symptomatic functioning based on a structured clinical interview.

## Materials and methods

### Participants

The study sample comprised *N* = 93 participants from the WARM study (Harder et al. 2015), of which 69 provided hair samples at pregnancy (*n* = 32 with severe mental illness (SMI), *n* = 37 non-clinical controls). Ethical approval for the WARM study came from Health Research Ethics, Capital Region of Denmark (protocol no: H-2-014-024). The study was preregistered in ClinicalTrials.gov Protocol Record DFF-1319-00103 (NCT02306551). Participants were recruited from obstetric wards in Region of Southern Denmark, the Capital Region, and Region Zealand, Denmark. Inclusion criteria were (1) women with first or subsequent pregnancies at the minimum age of 18 and (2) a diagnosis of either (a) lifetime DSM-5 delusional disorder, schizophreniform disorder, schizophrenia, schizoaffective disorder, psychosis NOS, and brief psychotic disorder, or (b) lifetime DSM-5 dipolar I or II disorder, or (c) a DSM-5 current or recurrent moderate-to-severe major depressive disorder, or (d) non-clinical controls. Exclusion criteria were (a) inability to provide informed and written consent to participate, (b) inability to speak English or Danish, (c) miscarriage, (d) maternal diagnosis of autism spectrum disorder, and (e) alcohol or drug dependency being the primary diagnosis. Further exclusion criteria were women describing current psychiatric symptoms not previously identified or treated and likely to require treatment.

### Procedure

Sociodemographic and clinical assessments were collected during 2nd or 3rd trimester shortly after recruitment. Maternal hair was collected in the 3rd trimester. For further information on study design, see Harder et al. (2015).

### Measures

#### Socio-demographics

Information on age, ethnicity, highest level of education, and civil status was self-reported. Education was categorized based on the ISCED-1997. All participants self-reported as married/living with a partner, or never married/not living with a partner, and civil status was dichotomized into “married or co-habiting”/not.

#### Clinical assessments

Psychopathology was measured by lifetime diagnosis of severe mental illness and current symptomatic functioning. Psychiatric diagnoses were confirmed using the psychosis and mood modules of the structured clinical interview (SCID) from the DSM-5 (First et al. 2016). All diagnostic assessments were supervised by a researcher trained on the SCID (KR, SH), and all diagnoses were confirmed through consensus discussion among the senior researchers. Based on the clinical interview, current symptomatic functioning was rated by the researcher using the symptomatic functioning subscale (GAF-S) of the global assessment of functioning scale (Niv et al. 2007). GAF is a numeric scale (0 through 100) with higher scores indicating better functioning. A score of 0 indicates insufficient information for rating, scores ranging from 10 to 20 indicate dangerous levels of symptoms present, and scores of 21–50 indicate dysfunctional levels of symptoms present. Scores of 51–70 indicate mild symptoms and some difficulty in functioning to serious symptoms and gross impairment in functioning. Scores above 71 indicate minimal symptoms and no more than slight impairment in functioning to no symptoms and superior functioning. Inter-rater reliability for the GAF-S subscale was acceptable (*ICC* (1) = 0.602).

#### Hair cortisol analyses

Hair samples were collected to determine cortisol concentrations. A section of hair strands approximately 3 mm in diameter was cut as close to the scalp as possible from a posterior vertex position. Hair samples were stored in aluminum foil as previously described by Anna-Hernandez et al. (2011) and analyzed at Dresden LabService GmbH, Germany, led by Prof. Dr. rer. nat. Clemens Kirschbaum, using liquid chromatography–tandem mass spectrometry (Gao et al. 2013). The 4-cm hair segment closest to the scalp was used for analyses to represent cortisol secretion over the most recent 4-month period (Stalder and Kirschbaum 2012). HCC values were standardized to the weight of the respective hair sample.

#### Confounding variables

Factors previously associated with maternal hair cortisol (Sauvé et al. 2007; Bublitz and Stroud 2012; Stalder and Kirschbaum 2012; Schreier et al. 2015; Marteinsdottir et al. 2021) were assessed as potential confounds, including (1) parity, (2) smoking, (3) regular use of medication, (4) body mass index (BMI), (5) waistline, (6) hair treatments over the past 3 months (dyeing, bleaching, permanent waves), (7) adverse childhood experiences, and (8) infant gender. Parity was categorized (yes/no) for primipara based on maternal self-report. Smoking was self-reported as number of cigarettes smoked in the past 7 days and categorized yes/no to smoking. Medication profile was self-reported as current use of any type of medication and categorized (yes/no). BMI was calculated by dividing weight (kg) by height squared (meter). Waistline was measured at the time of hair collection and reported in full cm. Use of chemical hair treatments over the past 3 months was self-reported at assessment and categorized (yes/no). Adverse childhood experiences were assessed using the self-report Adverse Childhood Experiences Study Questionnaire (ACES) (Felitti et al. 1998). The ACES has excellent internal reliability (Cronbach’s *α* = 0.88) (Murphy et al. 2013). Total number of adverse childhood experiences (ACE) was used as a continuous scale (range 0–10).

### Data analytic plan

All analyses were conducted using IBM SPSS Statistics 28. HCCs were normally distributed after Log10 transformation, and ANOVAs were used to model unadjusted differences on HCC between the SMI and control group. Skewness of the main variables symptomatic functioning (GAF-S) and square root transformed ACE were within the assumption of the rule of + 1 to − 1 (Auyeung et al. 2009). Skewness of demographic and confounding variables, including education, maternal age, marital status, primipara, smoking, BMI, medication use, waistline, hair treatment within the past 3 months, and gestational age at cortisol collection, did not improve with transformations. Non-parametric Kruskal–Wallis test was used on skewed continuous variables. Pearson’s **χ**^2^ was used for dichotomized variables, and Fisher’s exact test 2-sided significance was reported. Non-parametric Spearman’s correlations of main variables, demographic variables, and all confounders were performed to identify covariates in subsequent analyses. Variables correlating significantly with HCC were controlled for in subsequent regression models that were run to estimate the effect of SMI and GAF-S score on HCC. All categorical variables entered into the regression model were dummy coded; i.e., infant gender was coded 1 for boys with girls as reference, parity was coded 1 for primiparity and 0 for multiparity, smoking was coded 1 and 0 for not smoking, and having a lifetime diagnosis of severe mental illness was coded 1 and non-clinical controls 0.

## Results

### Sample description and identification of control variables

Table 1 presents sample characteristics by groups of SMI and controls. The total sample presented as low on socio-economic risks, and most women from both groups were married or living with a partner. There were significant differences between the two groups on education level, age, and total ACE in unadjusted analyses. Women with SMI differed from women in the control group on the GAF-S. Mean GAF-S score among women in the control group was 87.86, with no participants scoring below 70 (one participant scored 70), indicating absence of symptoms and good functioning. Mean GAF-S score among women with SMI was 68.06, indicating some mild symptoms and some difficulties in functioning. Kruskal–Wallis and Chi-square analysis showed that there were no significant differences between participants who did and did not provide hair samples on any of the main variables or demographic measures.

**Table 1:** Sample characteristics and distribution of main study variables (*N* = 69)

Severe mental illness (***N*** = 32)				Control (***N*** = 37)				
*Characteristics*	*N* (%)	*M*	SD	*N* (%)	*M*	SD	*H*(df)	*p*
Education: ≤ upper secondary education	22 (68.8)			30 (81.1)			F(1) = 15.86	< .001
Age (years)		29.94	4.16		33.13	4.39	*H*(1) = 7.23	.007
Married/living with partner	29 (90.6)			33 (89.2)				
Primipara	23 (71.9)			22 (59.5)				
Symptomatic functioning, GAF-S scale†		68.06	11.76		87.86	6.99		
≤ 70 on GAF ‡	18 (56.2)			1 (2.7)				
Total ACE§		2.48	2.00		1.00	1.41	*H*(1) = 8.12	.004
*Hair*								
Regular use of any medication (yes)	21 (65.6)			8 (21.6)				.035
Cigarette smoking	4 (12.5)			0 (0.0)				.113
Body mass index, kg/m^2^		30.95	5.23		27.36	3.97	*H*(1) = 9.00	.003
Gestational age (weeks)		35.59	3.03		36.00	2.13	*H*(1) = .03	.868
Color during last 3 months	3 (9.4)			2 (5.4)				.647
Maternal hair cortisol (pg/mg)** ¶**		9.64	6.72		4.95	3.54	*F*(1) = 12.78	< .001

^†^Assessed from the GAF during 3rd trimester of pregnancy

^‡^Scores equal to or below 70 on the GAF scale represent borderline, dysfunctional, or dangerous symptomatic levels of functioning

^§^Maternal adverse childhood experiences, total ACE, on the ACE questionnaire. Arithmetic mean, *p*-values on square root transformed variable

^¶^HCC arithmetic mean and SD, *p*-value after log10 transformation

Analysis of potential confounding variables, including (1) parity, (2) smoking, (3) regular use of medication, (4) BMI, (5) waistline, (6) hair treatments over the past 3 months, (7) total number of ACEs, and (8) infant gender, indicated that parity (*r* = 0.263, *p* = 0.030), education (*r* =  − 0.286, *p* = 0.027), BMI (*r* = 0.268, *p* = 0.028), and smoking (*r* = 0.268, *p* = 0.042) were significantly related to maternal HCC in pregnancy. These significant variables were included as control variables in subsequent multivariate regression models.

### Lifetime diagnosis of SMI as predictor of HCC

Unadjusted univariate ANOVA showed that HCC was significantly higher in women with SMI compared to controls (*F*(1) = 12.78, *p* =  < 0.001) (Table 1). A multivariate regression model was run to assess the effect of SMI on HCC, controlling for the relevant control variables (Table 2). Results showed that the association between SMI and HCC was significant with a small effect size (*β* = 0.221) when controlling for parity, education, BMI, and smoking (Table 2).

**Table 2:** Linear regression models of associations between severe mental illness and HCC in pregnancy controlling for parity, education, body mass index, and smoking

*Variables predicting HCC*	*β*	*CI*	*R*^*2*^ for model	*p-value*
Model			.345 F(5,45) = 4.743	.001
Primipara	.219	[.016, .423]		.035
Education	− .014	[− .116, .087]		.775
Body mass index	.011	[− .010, .032]		.287
Smoking	.214	[− .151, .579]		.244
Severe mental illness	.221	[.010, .432]		.041

Two-tailed *p*-value < .05. HCC = Log10 transformed maternal hair cortisol concentration in pregnancy. Smoking = smoking the past week. Severe mental illness of psychosis, bipolar disorder, or depression vs. control group

### Symptomatic functioning (GAF-S) as predictor of HCC

There was a significant correlation between GAF-S score and HCC (*r*_*s*_ (67) =  − 0.415, *p* =  < 0.001). To assess the robustness of this finding in relation to potential confounds, a multivariate regression was run with parity, education, BMI, and smoking included as control variables (Table 3). Results showed that the association between GAF-S and HCC remained significant with a small effect size (*β* =  − 0.011) (Table 3). As in the model with SMI, the control variable parity was significantly associated with HCC in this model with a medium effect size (*β* = 0.228) (Table 3).

**Table 3:** Linear regression models of associations between current symptomatic functioning and HCC in pregnancy controlling for parity, education, body mass index, and smoking

*Variables predicting HCC*	*β*	*CI*	*R*^*2*^ for model	*p-value*
Model			.375 F(5,43) = 5.161	< .001
Primipara	.228	[.022, .434]		.031
Education	.029	[− .087, .144]		.619
Body Mass Index	.014	[− .007, .036]		.193
Smoking	.317	[− .053, .686]		.091
GAF-S	− .011	[− .019, − .002]		.016

Two-tailed *p*-value < .05. HCC = Log10 transformed maternal hair cortisol concentration in pregnancy. Smoking = smoking the past week. GAF-S = global assessment of symptomatic functioning subscale score

## Discussion

We hypothesized that severe psychopathology, conceptualized as a lifetime diagnosis of SMI and current symptomatology, was associated with higher HCC. Our findings showed that both lifetime diagnosis of SMI and current symptomatology were significantly associated with HCC. In addition, parity accounted for significant variance in HCC levels.

This seems to be in accordance with previous findings. In their meta-analysis, Khoury et al. (2023) showed that the timing of maternal HCC and psychological distress measurements moderated the effects, such that studies that measured distress before HCC had significantly larger effects, compared to studies that measured HCC before distress, or distress and HCC concurrently. This could support our finding of a significant effect of lifetime diagnosis on HCC, as all participants in the SMI group in our study had been diagnosed prior to inclusion in the study.

We also found that poorer current symptomatic functioning was significantly associated with elevated HCC in pregnancy. This seems to contradict previous findings, as women in our sample did not present with high levels of current symptoms; i.e., mean GAF-S rating among women from the SMI group indicated some mild symptoms and some difficulties in functioning. Previous studies have reported no association between current prenatal symptoms and HCC in participants with overall low levels of psychological distress (Mustonen et al. 2018). In the large FinnBrain cohort sample (*N* = 595), Mustonen et al. (2019) found that the trajectories of depressive symptoms in pregnancy were significant in relation to HPA axis functioning. Mustonen found that the subgroup of mothers with “low and steeply increasing” depressive symptoms (low in early and midpregnancy, but high in end pregnancy) did not show elevated HCC postpartum. However, HCC measured at delivery was elevated in relation to continuously higher levels of depressive symptoms, and the mothers in the “consistently elevated” group presented with higher HCC than mothers in both the “consistently low” and the “low and increasing” groups (Mustonen et al. 2019). This could support the hypothesis that chronicity of the prenatal symptoms, rather than short-term symptomatology, is relevant when HPA axis functioning is investigated. Momentary or short-term states of stress might not significantly affect the long-term biological measures such as HCC. In turn, altered functioning of the HPA axis could also contribute to the persistence of psychological distress or symptoms. In our study, women describing current psychiatric symptoms not previously identified or treated was excluded. In addition, only women from the SMI group presented with ratings of clinical to sub-clinical levels of symptomatic functioning on the GAF-S compared to the control group. It could be argued that the effect of current symptomatic functioning on HCC found in this sample is an effect of current symptomatology in concomitant of a lifetime diagnosis of severe mental illness. Thus, our sample may represent women who have had more chronic exposure to psychological distress over a lifetime, and a more continuous strain on the HPA axis, compared to women in the non-clinical control group, although some women from the SMI group did not present with high levels of current symptoms at the time of inclusion.

Studies reporting a positive association between prenatal psychological distress and HCC appear to have a larger variance in psychological distress symptom severity (Mustonen et al. 2018). Thus, our findings of significant effects of the constructs of lifetime diagnosis as well as current symptomatology on HCC in pregnancy may be partly explained by our sample characteristics including greater variance in psychiatric symptoms and higher frequency of lifetime distress symptoms.

Finally, short-term stress might not significantly affect the offspring risks related to prenatal psychological distress. Thus, identifying situations with different risk profiles would enable further understanding of the mechanisms linking prenatal maternal psychological distress to adverse offspring outcomes (Mustonen et al. 2018). In our study, both lifetime diagnosis of moderate-to-severe depression, bipolar disorder, or schizophrenia spectrum disorders and current symptomatic functioning predicted maternal HCC in late pregnancy and remained significant with other potential confounding variables controlled. This raises compelling concerns for the risks to both mother and infant of heightened glucocorticoids during this sensitive period of early development. In a previous study using a smaller subsample of the same participants from the WARM study, we found that maternal HCC in pregnancy and at 4 months postpartum mediated associations between maternal psychopathology and maternal disrupted behavior in interaction with the infant, both when psychopathology was measured as lifetime diagnosis and as current symptomatic functioning (Nyström-Hansen et al. 2019). These findings indicate that HCC may be a potential early biomarker for future caregiving challenges. Despite the difficulties in recruiting samples of women with severe mental illnesses during the limited timespan of pregnancy, our results clearly indicate the value of future studies with larger sample sizes to assess the replicability of the results reported here.

### Limitations

Some limitations of the study should be noted. First, the small sample size limits the power to analyze potential differences between diagnostic groups. Second, this study did not include measures of other stressful life events or frequency of hair washing, which could affect HCC (Stalder et al. 2017). In addition, it could be noted that our measure of hair treatments (dyeing, bleaching, permanent waves) only asked about the past 3 months, though segments of 4 cm hair were analyzed reflecting the prior 4 months. However, in the recent study by Mustonen et al. (2019) on 595 participants from the large FinnBrain Birth Cohort Study, the researchers did not find HCC to be associated with hair-related attributes such as hair dying or hair washing. Similarly, meta-analytic estimates (Stalder et al. 2017) concerning the influences of hair washing frequency, and hair treatment suggested that although it is good practice to adjust for these factors, under most circumstances their influences would be practically negligible. Accordingly, we did not find a correlation between HCC and hair treatment.

## Conclusions

Having a diagnosis of schizophrenia spectrum disorder, bipolar disorder, or current/recurrent moderate-to-severe major depressive disorder at some point prior to becoming pregnant predicts elevated maternal HCC during pregnancy. In addition, poorer current symptomatic functioning is associated with elevated HCC in pregnancy, compared to controls. The implications of elevated HCC on both maternal and infant health need further study. Hair cortisol concentration may be a potential early biomarker for developmental risk.
